# On the Utility of Medicated Frictions in Obstinate Disorders

**Published:** 1799-03

**Authors:** 

**Affiliations:** Pavia


### *On the Utility of medicated Frictions in obstinate Disorders:— By Professor* Brera, *of Pavia.*

It is an admitted fact, that medicinal substances may be introduced into the human system through the pores, by means of the lymphatic or absorbent vessels; and that this mode of applying powerful medicines, without any co-operation on the part of the digestive organs, has been attended, in a variety of instances, with many and singular advantages. Is it not, therefore, matter of surprize, that this simple and beneficial process, with which it appears the ancients were familiarly acquainted, and which they continually practised in their medicated baths, should have become almost totally neglected by the moderns, if we except mercurial frictions in cases of lues?

Professor Brera, convinced by a number of experiments of the superior efficacy, which the external application of different medical substances possesses over that of burthening the stomach with heterogeneous compounds, has lately published a third edition of his "*Programma del modo d'agire sul corpo umano per mezzo di frizioni fatte con salive, &c.**" in which he communicates some judicious observations derived from his own experience on this important subject, together with the result of the inquiries of other respectable physicians in Italy. According to the former of these, which he had frequent opportunities to repeat on different patients, afflicted with similar disorders, he acquaints us, that, for instance in the gout, he has successfully employed frictions with an ointment compounded of the *aconitum napellus* Lin. opium, and saliva; in dropsical cases, he has recommended with equal benefit a mixture of the squil, or digitalis and saliva; to febrile patients, whose *primæ viæ* were obstructed, he directed the external application of the antimonium tartar, freely blended with saliva, or any animal fat, as hog's-lard, &c; and thus (adds the Professor) the causes generating these diseases were sooner or later happily removed.

We shall just observe here, that our expectations are not very sanguine respecting the success likely to attend any *new* remedies or methods of cure, which a fertile invention may yet contrive: from deliberate conviction, we are of opinion, that the practice of medicine will be greatly more benefited and improved by an accurate diagnosis and nosological description of diseases (in both of which we come short perhaps of the ancients), than by the most promising discoveries in the Materia Medica, and the doctrine of therapeutics.—As it appears, however, that Professor Brera's suggestions and experiments

* The learned Professor has also reprinted the *second* edition of this interesting practical essay in the "*Commentarii Medici*," of which he is the editor.

experiments have been adopted and pretty extenſively followed up by ſeveral gentlemen who rank high as Italian phyſicians, and of whom we ſhall only mention the names of BENVENUTI, LOCATELLI, and BOTELLI, who have directed ſimilar medicated frictions in caſes of gout and dropſy, as alſo in diſorders accompanied with acute pain between the falſe ribs, convulſive cough, &c. and that they have completely cured, or at leaſt conſiderably relieved the ſituation of ſuch patients; we are induced to recommend this method to the attentive inveſtigation and trial of the medical practitioner in Britain.

The ingenious Profeſſor concludes with the following obſervation, that medicated frictions will in all probability be found moſt ſuccefsful in the treatment of diſeaſes to which youth and infancy are ſubject, in caſes of hydrophobia, &c.—for the corroboration of which, however, we do not find that he refers to any practical inſtances.

